# Supplementary material for: Mixed trends in heavy metal-enriched fugitive dust on National Park Service lands along the Red Dog Mine haul road, Alaska, 2006–2017
Source: PLoS One. 2024 Feb 27;19(2):e0297777. doi: 10.1371/journal.pone.0297777 (PMC10898755; doi:10.1371/journal.pone.0297777)
Supplement: Attachment — Submitted filename: Response to Reviewers.docx [file pone.0297777.s001.docx]

**Response to Reviewers**

From: Peter Neitlich, National Park Service

To: Dr. Maurizio Fiaschetti, Academic Editor, PLOS ONE

Re: Response to Reviewers

Date:

Dear Dr. Fiaschetti,

Thank you for forwarding the excellent suggestions for revisions to this manuscript. These comments have helped to create a much better version of the final product. I have responded to each revision suggestion and have summarized resulting revisions in the comment matrix below. I am confident that we captured the essence of the requested revisions, but please do let me know if you still see any unresolved issues.

Best wishes,

Peter Neitlich

| **Reviewer** | **Comment** | **Revision** |
| --- | --- | --- |
| **Academic Editor** |  |  |
| Academic Editor | 1. Please ensure that your manuscript meets PLOS ONE's style requirements,  including those for file naming. The PLOS ONE style templates can be found at  https://journals.plos.org/plosone/s/file?  id=wjVg/PLOSOne_formatting_sample_main_body.pdf and  https://journals.plos.org/plosone/s/file?  id=ba62/PLOSOne_formatting_sample_title_authors_affiliations.pdf | Completed. |
| Academic Editor | 2. Please note that PLOS ONE has specific guidelines on code sharing for  submissions in which author-generated code underpins the findings in the  manuscript. In these cases, all author-generated code must be made available  without restrictions upon publication of the work. Please review our guidelines  at https://journals.plos.org/plosone/s/materials-and-software-sharing#loc-sharingcode  and ensure that your code is shared in a way that follows best practice and  facilitates reproducibility and reuse. | Completed. |
| Academic Editor | 3. Thank you for stating the following in the Acknowledgments Section of your  manuscript:  “This study was funded by the National Park Service’s Arctic Network (Inventory and  Monitoring Program).” We note that you have provided additional information within the Acknowledgements Section that is not currently declared in your Funding  Statement. Please note that funding information should not appear in the Acknowledgments section or other areas of your manuscript. We will only publish  funding information present in the Funding Statement section of the online submission form. Please remove any funding-related text from the manuscript and let us know how  you would like to update your Funding Statement. Currently, your Funding Statement reads as follows:  “This research was funded by the National Park Service, Arctic Network (Inventory and Monitoring Program).” Please include your amended statements within your cover letter; we will change the online submission form on your behalf. | We have removed all references to funding from the manuscript. The funding statement (“This research was funded by the National Park Service, Arctic Network (Inventory and Monitoring Program).”) currently reads correctly and is not in need of revision. |
| Academic Editor | 4. Thank you for stating the following financial disclosure:  “This research was funded by the National Park Service, Arctic Network (Inventory  and Monitoring Program).”  Please state what role the funders took in the study. If the funders had no role,  please state: "The funders had no role in study design, data collection and analysis,  decision to publish, or preparation of the manuscript."  If this statement is not correct you must amend it as needed.  Please include this amended Role of Funder statement in your cover letter; we will  change the online submission form on your behalf. | Please add the following statement: "The funders had no role in study design, data collection and analysis, decision to publish, or preparation of the manuscript." |
| Academic Editor | 5. Please include your full ethics statement in the ‘Methods’ section of your  manuscript file. In your statement, please include the full name of the IRB or ethics  committee who approved or waived your study, as well as whether or not you  obtained informed written or verbal consent. If consent was waived for your study,  please include this information in your statement as well. | An ethics statement was added. |
| Academic Editor | 6. We note that Figures 1, 3, 4, 5, 6, 7, 8, 9, 10, 11, 12, 13 and 14 in your  submission contain map images which may be copyrighted. All PLOS content is  published under the Creative Commons Attribution License (CC BY 4.0), which  means that the manuscript, images, and Supporting Information files will be freely  available online, and any third party is permitted to access, download, copy,  distribute, and use these materials in any way, even commercially, with proper  attribution. For these reasons, we cannot publish previously copyrighted maps or  satellite images created using proprietary data, such as Google software (Google  Maps, Street View, and Earth). For more information, see our copyright  guidelines: <http://journals.plos.org/plosone/s/licenses-and-copyright>.  We require you to either (1) present written permission from the copyright holder to  publish these figures specifically under the CC BY 4.0 license, or (2) remove the  figures from your submission:  a. You may seek permission from the original copyright holder of Figures 1, 3, 4, 5,  6, 7, 8, 9, 10, 11, 12, 13 and 14 to publish the content specifically under the CC BY  4.0 license.  We recommend that you contact the original copyright holder with the Content  Permission Form (http://journals.plos.org/plosone/s/file?id=7c09/content-permissionform.  pdf) and the following text:  “I request permission for the open-access journal PLOS ONE to publish XXX under  the Creative Commons Attribution License (CCAL) CC BY 4.0  (http://creativecommons.org/licenses/by/4.0/). Please be aware that this license  allows unrestricted use and distribution, even commercially, by third parties. Please  reply and provide explicit written permission to publish XXX under a CC BY license  and complete the attached form.”  Please upload the completed Content Permission Form or other proof of granted  permissions as an "Other" file with your submission.  In the figure caption of the copyrighted figure, please include the following text:  “Reprinted from [ref] under a CC BY license, with permission from [name of  publisher], original copyright [original copyright year].”  b. If you are unable to obtain permission from the original copyright holder to  publish these figures under the CC BY 4.0 license or if the copyright holder’s  requirements are incompatible with the CC BY 4.0 license, please either i) remove  the figure or ii) supply a replacement figure that complies with the CC BY 4.0  license. Please check copyright information on all replacement figures and update  the figure caption with source information. If applicable, please specify in the figure  caption text when a figure is similar but not identical to the original image and is  therefore for illustrative purposes only.  The following resources for replacing copyrighted map figures may be helpful:  USGS National Map Viewer (public domain): http://viewer.nationalmap.gov/viewer/  The Gateway to Astronaut Photography of Earth (public  domain): http://eol.jsc.nasa.gov/sseop/clickmap/  Maps at the CIA (public domain): https://www.cia.gov/library/publications/the-worldfactbook/  index.html and https://www.cia.gov/library/publications/cia-mapspublications/  index.html  NASA Earth Observatory (public domain): http://earthobservatory.nasa.gov/  Landsat: http://landsat.visibleearth.nasa.gov/  USGS EROS (Earth Resources Observatory and Science (EROS) Center) (public  domain): http://eros.usgs.gov/#  Natural Earth (public domain): http://www.naturalearthdata.com/ | The hillshade map presented in Fig 1 was created from the USGS National Elevation Dataset. As stated in the attached document USGS NED, all USGS National Elevation Dataset data are public domain and may be used freely without copyright permission. Documentation attached.  The spatial data layer containing the boundary for Cape Krusenstern National Monument and used in Figures 3,4,5,6,7,8,9,10,11,12,13 and 14 are public domain and available for download from the USGS National Map Viewer. This layer contains no imagery, simple a GIS polygon feature created by the U.S. Government for public use. |
| **Reviewer 1** |  |  |
| Reviewer 1 | I have revised the manuscript PONE-D-23-00195 and I found that this work lack of a  well-documented and properly supported hypothesis. Data are just too much  descriptive and the weight of the entire manuscript relays merely in statistics. | This criticism is often made of long-term monitoring studies. By definition, long-term monitoring—a vitally important activity to ensure natural resource protection—is driven by research on status and trends rather than any distinct hypothesis. Insertion of a hypothesis of “no change” is certainly possible, but would appear overly obvious and *pro forma* to most readers.  PLOS One’s requirements are: “Primary research that contributes to the base of scientific knowledge, including interdisciplinary, replication studies, and negative or null results.”  I would point the editor to this paper (<https://www.ncbi.nlm.nih.gov/pmc/articles/PMC8207363/>) which documents that the use of formal hypotheses in ecological and evolutionary papers occurs in only 6-25% of publications.  In order to clarify the status of this paper as a long-term monitoring study, we have added text to the Abstract and Introduction about long-term monitoring programs and our program in particular. |
| Reviewer 1 | No other kind of discussion regarding the extremely important biological and  environmental factors that for sure are impacting in the level of metals detected in  the studied area are even proposed. For example, biogenic sources of metals, the  yield of moss biomass during time, the impact of climate change in moss physiology  and at the studied area (e.g., rainfall rate, rainfall intensity, sun irradiation, etc.), the  life cycle of these specimens, the duplicating time, the viability and physiological  affection due to heavy metals accumulation, type of sediments, quality of soil, etc.  etc. etc.… none of these aspects (or at least some of them) are considered in this  manuscript. Therefore, without any other aim more than the statistics itself, from my  point of view, conclusions are not meaningful at all. I am truly sorry, but in its  present form I cannot recommend this article to be publish in PLOS ONE. | Again, this paper represents the third installment of long-term monitoring that began in 2001 and which are cited in this manuscript. Earlier papers did consider alternate sources of metal distribution, the suitability of *Hylocomium splendens* as a biomonitor in this study area, and metal concentrations in peat soils and unaffected parent material.  From a broader perspective, three main points are important here:   1. This paper reports on the results of published long term monitoring protocols, and these methods (including sampling design, field work, and statistics) are designed to be fixed through time to ensure repeatability. 2. This paper is based on methods already published twice in this journal, and in several other journal articles cited in the manuscript. 3. The use of biomonitoring via the elemental analysis *Hylocomium splendens* tissue is well-established in the literature and has been used in this fashion for over 40 years. While there are certainly debates about global comparisons of moss biomonitoring data based on the types of climate-driven physiology mentioned by Reviewer 1, the use of these methods for contaminant status and trends analysis in regional and fine-scale studies has been upheld by dozens of peer-reviewed publications and review articles.   We have added some background on the widespread use of this method (Lines 65-82). It is, however, far beyond the scope of this monitoring study to re-investigate all of the physical processes that may influence the utility of moss biomonitoring in our protocol. |
| **Reviewer 2** |  |  |
| Reviewer 2 | The paper prepared by Peter N. Neitlich and co-authors looks like a  report and not a scientific paper. Usually, scientific papers are not written in the first  person! | We respectfully disagree with Reviewer #2 on first person usage. Most science writing has migrated towards first person usage in journal articles to promote accuracy and simplicity. A simple glance through the high-profile journals Science or Nature will confirm this. Oxford University Press recommends this voice to reduce ambiguity and promote brevity: <https://blog.oup.com/2018/01/first-person-pronouns-passive-voice-scientific-writing/>. Here’s a random example pulled from the journal Science today: <https://www.science.org/doi/10.1126/science.abg2482>. |
| Reviewer 2 | All parts of the manuscript need to be significantly improved.  1. Introduction: The novelty of the study needs to be added. Study is of very local  interest, how the results can be used by international community? | We have addressed the novelty of results by adding language about the uses of this paper for studying mineral ore dusts rather than the more common literature on smelting effects. This paper has broad application anywhere mining or ore transport is occurring, and many in Alaska are concerned about the likelihood of contaminants issues as the Ambler mine progresses towards initiation. Nonetheless, it should be stated that the utility of results by the international community is not a criterion for publication in PLOS One. [Journal Information \| PLOS ONE](https://journals.plos.org/plosone/s/journal-information#loc-criteria-for-publication). |
| Reviewer 2 | 2. Methods: how many samples were collected for analysis, which techniques were  used to determine elements concentration of the elements? | As mentioned in the paper, the methods including sample numbers are unchanged from the previous publication in this journal. However, since many readers won’t reference these earlier publications, we have provided a skeletal version of the methods here and have included a new table with 2017 samples that has 6 more samples than the 2006 sampling. |
| Reviewer 2 | 3. Results and Discussion: in the manuscript there presented results, but there is no  discussion | We have added discussion of biological effects and mitigation. |
|  |  |  |
| **Reviewer 3** |  |  |
| Reviewer 3 | I found your manuscript to be well-written and concise. However, I suggest that you  provide more details on the sampling and laboratory analyses methods used, to add  to the clarity of the manuscript. | We have added details on sampling. Lab analysis is referenced and available open source from the previous PLOS One publication on this monitoring program. |
| **Reviewer 4** |  |  |
| Reviewer 4 | The authors presented the results of a decadal spatial analysis of  contaminant deposition based on the resampling of locations Cape Krusenstern  National Monument (CAKR), Alaska, adjacent to the Red Dog Mine haul road.  There are several comments to be solved by the authors, after that I can  recommend its publication in PLOS ONE.  - The purpose of this study is not very clear stated in the abstract. Please add the  importance of your findings also. | We have clarified the purpose of this study in the abstract: to report on the status and trends of long-term monitoring of heavy metal deposition via moss biomonitoring in Cape Krusenstern National Monument, Alaska. |
|  | Please describe more in Field and Lab Methods how many samples did you  collect. How did you keep these until the analyses? What is the unit of measurement  for your assays? | We provided a table of samples by distance classes, and briefly described storage methods in Methods section. The units of measurement (mg/kg of element in moss tissue dry weight) were added to methods, and were already present in Results and Discussion in Tables 3, 4, 5. Our reporting of mg/kg dry weight of elements in moss tissue is standard for biomonitoring. |
|  | I suggest to comment your results in comparison with other available studies in this domain of research, and related to the human activities organized in that region.  The paper has no discussions in this moment. | We have added content in both the Introduction and Results and Discussion putting the results in context of other related studies. As far as we are aware, this monitoring program is the only one tracking mining fugitive dusts (which we have noted), so comparisons are more limited than for other areas of mining-related pollution. |
|  | Regarding your last phrase in the Conclusions, did you measure the lichen  recovery? Can you support your statement? | We did not measure lichen recovery, though Di Meglio’s cited M.S. Thesis concluded that lichen species richness was continuing to decrease. We have explained that recovery is theoretically possible where deposition has dropped below proposed effects thresholds, but certainty on this will require ongoing study of lichen communities and contaminants. We also state above that the proposed threshold is preliminary and itself requires more study. |
