# Supplementary material for: Mixed trends in heavy metal-enriched fugitive dust on National Park Service lands along the Red Dog Mine haul road, Alaska, 2006–2017
Source: PLoS One. 2024 Feb 27;19(2):e0297777. doi: 10.1371/journal.pone.0297777 (PMC10898755; doi:10.1371/journal.pone.0297777)
Supplement: Attachment — Submitted filename: Response to Reviewers.docx [file pone.0297777.s002.docx]

**Response to Reviewers**

From: Peter Neitlich, National Park Service

To: Dr. Maurizio Fiaschetti, Academic Editor, PLOS ONE

Re: Response to Reviewers

Date:

Dear Dr. Fiaschetti,

Thank you for forwarding the excellent suggestions for revisions to this manuscript. These comments have helped to create a much better version of the final product. I have responded to each revision suggestion and have summarized resulting revisions in the comment matrix below. I am confident that we captured the essence of the requested revisions, but please do let me know if you still see any unresolved issues.

Best wishes,

Peter Neitlich

| **Reviewer** | **Comment** | **Revision** |
| --- | --- | --- |
| **Academic Editor** |  |  |
| Academic Editor | I encourage the authors to address the issues raised by Reviewer 5 and in particular, I encourage them to provide a more thorough discussion of the methodology used and the results obtained. | We have greatly enhanced the methods section and have also pointed readers who want more information to open source publications that report methods of this long-term monitoring protocol in granular detail. |
| Academic Editor | All the abbreviations need to be explained too. | Completed. |
|  |  |  |
| **Reviewer 5** | Your manuscript seems interesting because it raises an important environmental issue. The layout of the manuscript is standard for scholarly articles. The introduction and purpose were written correctly. Unfortunately, the next chapters need improvement. The methodology is not clear and does not reflect the Results and Discussion chapter. | We have expanded the methods section and have also pointed readers who want more information to open source publications in this journal that report methods of this long-term monitoring protocol in high detail. We have also explained our goals in the methods better such that they align with the Results and Discussion. |
|  | There is no explanation of the abbreviations under the tables. | All abbreviations have now been defined in each table legend. |
|  | The discussion of the results is cursory and  general. | We have considerably enhanced the discussion of biological effects and have discussed analogous literature on recolonization of lichens following pollution reduction. |
|  | Literature should not be cited in the conclusion. | We have moved this idea and citation to the Results and Discussion section. |
|  |  |  |
